# Evaluating the Efficacy of a Multidisciplinary Approach to Chronic Pharyngitis Treatment: A Retrospective Cohort Analysis

**DOI:** 10.7759/cureus.80772

**Published:** 2025-03-18

**Authors:** Oguz Guvenmez, Huseyin Keskin, Anara Keneshovna Zhanbaeva

**Affiliations:** 1 Otolaryngology - Head and Neck Surgery, Osh State University, Osh, KGZ; 2 Otorhinolaryngology, Private Mersin Su Hospital, Mersin, TUR; 3 Clinical Pharmacology, Osh State University, Osh, KGZ

**Keywords:** chronic pharyngitis, comprehensive treatment strategy, multidisciplinary approach, quality of life, sore throat quality of life scale

## Abstract

Background: Chronic pharyngitis (CP) presents a significant burden on patients' quality of life (QoL), often requiring comprehensive management strategies beyond conventional treatment modalities. This study aims to evaluate the efficacy of a multidisciplinary approach to CP treatment and its impact on patients' QoL.

Methods: We conducted a retrospective analysis of 140 patients diagnosed with CP who were treated at the ENT outpatient clinic of Private Mersin Su Hospital, Mersin, Türkiye, between January 1, 2022, and January 1, 2023. Our treatment approach integrated dietary modifications, management of gastroesophageal reflux, nasal obstruction correction, and allergy management alongside standard pharmacotherapy. The primary outcome measure was the improvement in the Sore Throat QoL Scale (STQoL) scores, assessed at baseline, 30, 60, and 90 days post treatment.

Results: The study population consisted of 140 chronic pharyngitis patients, including 82 male (58.57%) and 58 female (41.43%) patients, with a mean age of 42.2 ± 6.3 years. Laboratory tests revealed that 115 patients (82.14%) had iron deficiency with a mean serum level of 61.2 mg/dL (reference range: 70-180 mg/dL), while 109 patients (77.85%) had low ferritin levels (mean 55.3 mcg/L; reference range: 50-150 mcg/L). In addition, 98 patients (70.00%) showed vitamin B12 deficiency, averaging 223.6 pg/mL (reference range: 350-900 pg/mL), 128 patients (91.42%) had vitamin D deficiency, with a mean value of 29.3 ng/mL (reference range: 40-100 ng/mL), and 68 patients (48.57%) demonstrated folic acid deficiency, with an average of 6.9 ng/mL (reference range: 6-17 ng/mL). Moreover, elevated glycated hemoglobin (HbA1c) levels were observed in 59 patients (42.14%) (mean 6.1%, target: 4-5.6%), and 45 patients (32.14%) had high serum total immunoglobulin E (mean 95.4 mg/dL; normal range: 20-100 mg/dL). Patient-reported outcomes using the STQoL improved markedly from a baseline score of 22.2 ± 3.2 to 57.9 ± 8.2 on day 30, 77.1 ± 13.6 on day 60, and 93.5 ± 17.3 on day 90 (all comparisons p < 0.05), indicating significant enhancement in QoL over the treatment period.

Conclusion: The findings suggest that a multidisciplinary approach to CP treatment, addressing both the symptoms and the underlying causes, significantly improves patients' QoL. This study underscores the importance of adopting comprehensive, patient-centered treatment strategies in managing chronic pharyngitis, warranting further research into optimizing care for this common condition.

## Introduction

Chronic pharyngitis (CP) is an extensively observed condition within the upper respiratory tract that impacts individuals irrespective of age or demographic. This ailment is distinguished by the inflammation of the pharyngeal mucosa, attributed to either microbial or non-microbial instigators [1]. The annual incidence rate has been documented at 14% among adults and an even higher rate of 41% in children [2], suggesting a significant public health concern. Notably, these figures may underrepresent the true prevalence of CP, as cases managed by general practitioners are not always recorded in specialized healthcare statistics.

Individuals suffering from CP often experience a spectrum of symptoms that detrimentally affect their daily life. These symptoms include, but are not limited to, a persistent sore throat, sensations of tickling or itching, stinging pain, disruptions in sleep patterns, post-nasal drip, and a feeling of constriction or a "lump" in the throat, along with persistent coughing. The severity of these symptoms can markedly diminish the affected individuals' quality of life (QoL) [3]. Complications may extend to difficulty in swallowing and, in cases where the inflammation affects the vocal cords or larynx mucosa, may result in vocal hoarseness or disorders. Furthermore, certain occupational environments that necessitate prolonged vocalization or exposure to pollutants such as dust and smoke are identified as risk factors for CP.

In addition to occupational exposures, the etiology of CP encompasses a range of factors. Notably, gastroesophageal reflux has been implicated as a causative factor, where the nocturnal ascent of stomach acids into the pharynx leads to mucosal damage and subsequent irritation [4,5]. The disease course can be further exacerbated by conditions affecting the cardiovascular system, the female genital tract, cervical osteochondrosis, and sleep apnea, thereby perpetuating the inflammatory state within the nasopharynx [6].

Pathophysiologically, CP is characterized by the incursion of chronic inflammatory elements, including macrophages and lymphocytes, into the submucosal layer following initial mucosal damage caused by viral, bacterial, or chemical agents [6,7]. This inflammation can extend to the tubopharyngeal ridges, often manifesting as pain that radiates towards the ears [8,9]. Pharyngoscopic examination typically reveals signs of hyperemia across the posterior pharyngeal wall and palatine arches, with visible inflamed lymphoid granules and potential tonsillar hyperplasia [7]. Notably, the typical symptoms associated with tonsillitis may not be present in CP cases. Moreover, CP can serve as an early indicator of various infectious diseases, including measles, scarlet fever, and rubella.

The progression and severity of CP are significantly influenced by the nature and virulence of the microbial flora, the extent of contamination, the overall health of the host organism, and the integrity of the mucosal membrane. Local immunity factors, such as innervation, hydration, and blood circulation within the mucosal tissues, also play critical roles [9,10]. Interestingly, while pain in pharyngitis and during exacerbations of CP is primarily due to the pharynx's rich innervation, this same anatomical feature can lead to the referral of pain to the ear and lower jaw. Despite extensive investigation, the precise role of bacterial or viral agents in the etiology of CP remains elusive, with diagnosis predominantly based on clinical observation and patient-reported symptoms [7].

Given the complex interplay of factors contributing to CP, this study aims to explore the outcomes of a multidisciplinary approach to treatment. By comparing these outcomes with those derived from conventional treatment modalities, our objective is to ascertain the efficacy and potential advantages of a comprehensive treatment strategy in managing CP, thereby enhancing patient care and QoL. This initiative reflects our commitment to advancing clinical practice through evidence-based interventions and underscores the importance of addressing CP with a nuanced understanding of its multifactorial etiology.

## Materials and methods

This was a retrospective study of a cohort of 140 patients diagnosed with chronic pharyngitis at the ENT outpatient clinic of Private Mersin Su Hospital, Mersin, Türkiye, between January 1, 2022, and January 1, 2023. The study protocol and all associated procedures were reviewed and approved by the Institutional Review Board (IRB) of Private Mersin Su Hospital, ensuring compliance with ethical standards and patient safety considerations. The IRB approval underscored our commitment to upholding the highest ethical standards in conducting this research, emphasizing the importance of patient consent, confidentiality, and the ethical treatment of all participants involved in the study. The study was reviewed and approved by the Ethics Committee of Niğde Ömer Halisdemir University (document number: 05/04/2023-342305).

Patients eligible for inclusion had a documented history of chronic pharyngitis symptoms such as stinging, burning, or tickling in the throat, persisting for more than a month. Comprehensive records detailing patient demographics, clinical presentations, treatment regimens, and follow-up outcomes were meticulously compiled.

The clinical evaluation involved a thorough examination of the pharyngeal anatomy using a flexible naso-pharyngo-laryngoscope, focusing on key areas including the tonsils, posterior pharyngeal wall, soft palate, tongue base, vallecula, pyriform sinuses, and laryngeal structures such as the glottis and supraglottic areas.

All chronic pharyngitis patients in this study received a multifaceted treatment approach. Based on clinical findings, a multidisciplinary strategy was employed, taking into consideration nasal pathology, the presence of gastroesophageal reflux, allergies, and vitamin-mineral deficiencies.

Following the initial clinical assessment, patients underwent a series of routine hematological and biochemical tests, assessing parameters such as complete blood count, blood sugar, urea, creatinine, and a comprehensive serology profile. Additional tests specific to this patient group included a thyroid function panel, liver function tests, and quantification of serum iron, ferritin, vitamin B12, vitamin D, and folic acid levels. For participants aged over 40, glycated hemoglobin (HbA1c) levels were measured, alongside serum total immunoglobulin E (IgE) to evaluate metabolic function and immune status.

Study tool

The Sore Throat QoL (STQoL) scale is a metric to gauge the impact of chronic pharyngitis on patients' daily lives [11]. This validated tool, tailored to the Turkish-speaking population within our clinic, comprises a 21-question survey to assess social, physical, and environmental aspects of living with pharyngitis or tonsillitis (Table 1). In order to assess the impact of chronic pharyngitis on daily life, the STQoL scale was administered to all patients at baseline and during follow-up visits on the 30th, 60th, and 90th day after treatment. Each question is scored on a five-point Likert scale ranging from “not at all” (5) to “extremely” (1), with higher scores indicating better QoL.

**Table 1:** Sore Throat Quality of Life (STQoL) scale Note: The responses are rated from 1 to 5 on a Likert scale Source: Catic et al., 2018 [11]; available under the terms of the Creative Common Attribution-NonCommercial 4.0 International (CC BY-NC 4.0)

Questions
How much your throat hurts, i.e. “burns” or “scratches”?
Does the sore throat make swallowing difficult?
How much does the sore throat affect your sleep?
Does the sore throat make breathing difficult?
Do you feel exhausted due to the sore throat?
Are you able to take care of yourself completely?
Do you walk less due to the sore throat?
How much does the sore throat affect your concentration?
Does the sore throat make you depressed?
Does the sore throat interfere with your daily religious activities?
Does the sore throat affect your relations with family members?
Does the sore throat interfere with your work?
Does the sore throat make following media more difficult?
Does the sore throat interfere with your friendships?
Does the sore throat interfere with your sexual activities?
Does the sore throat make your colleagues at work uncomfortable?
Do you have financial losses due to the sore throat?
Does the sore throat impair your personal security?
Does your sore throat prevent others from socializing with you?
Does the sore throat impair your ability to withstand air pollution in the city?
Does the sore throat impair your ability to withstand heat or coldness?

Data collection and analysis

Data collection intervals were set at the 30th, 60th, and 90th days post initial treatment to monitor the evolution of patient-reported outcomes. The STQoL scale scores were actively collected for the purpose of this study to ensure accuracy and consistency in follow-up intervals. Scores from the STQoL scale were aggregated and analyzed, comparing pre-treatment and post-treatment responses to determine the efficacy of the interventions employed. All statistical analyses, including the calculation of mean scores and the determination of percentages, were conducted using Microsoft Excel (Microsoft Corporation, Redmond, Washington, United States), providing a robust framework for the systematic evaluation of our multidisciplinary treatment approach.

## Results

The cohort included 140 chronic pharyngitis patients, consisting of 82 male (58.57%) and 58 female (41.42%) patients, aged 42.2 (± 6.3 SD) years on average. Blood tests showed results in line with the international standard reference values and revealed information with respect to their nutritional status and inflammation markers. Table 2 shows the nutritional deficiencies and metabolic markers of the cohort. Of the 140 participants, 115 (82.14%) patients were suffering from iron deficiency, with a mean serum level of 61.2 mg/dL, below the standard reference range of 70-180 mg/dL. Ferritin levels, reflecting the body's iron stores, were below the normal range of 50-150 mcg/L in 109 patients (77.85%), with a mean of 55.3 mcg/L. Vitamin B12 deficiency was registered among 98 (70%) patients with a mean of 223.6 pg/mL against an appropriate range of 350-900 pg/mL. Vitamin D was also significantly deficit in 128 (91.42%) subjects with a mean of 29.3 ng/mL way, below the reference of 40-100 ng/mL. Folic acid deficiency was noted in 68 patients (48.57%), with a mean level of 6.9 ng/mL, which is below the reference range of 6-17 ng/mL. Glucose dysregulation, as indicated by elevated HbA1c levels, was found in 59 patients (42.14%), with an average of 6.1%, exceeding the normal range of 4-5.6%. Furthermore, elevated serum total immunoglobulin E (IgE) levels were detected in 45 patients (32.14%), with an average of 95.4 mg/dL, surpassing the normal range of 20-100 mg/dL. These findings underscore the high prevalence of nutritional deficiencies and metabolic irregularities in patients with chronic pharyngitis.

**Table 2:** Prevalence and mean levels of nutritional deficiencies and elevated metabolic markers in the participants (N=140)

Parameters	Frequency	Percentage	Mean Value	Reference Range
Iron deficiency	115	82.14	61.2	70-180 mg/dL
Ferritin deficiency	109	77.85	55.3	50-150 mg/L
Vitamine B12 deficiency	98	70	223.6	350-900 ng/mL
Vitamin D deficiency	128	91.42	29.3	40-100 ng/mL
Folic acid deficiency	68	48.57	6.9	6-17 ng/mL
Elevated HbA1c	59	42.14	6.1	%4.7-5.6
Elevated lg E	45	32.14	95.4	20-100 mg/dL

In terms of patient-reported outcomes measured using the STQoL scale, significant improvements were observed post treatment. The initial mean STQoL score was 22.2 (± 3.2 SD). Following the intervention mentioned in the method section, there was a considerable increase in the STQoL scores on the 30th day (57.9 ± 8.2), 60th day (77.1 ± 13.6), and 90th day (93.5 ± 17.3) evaluations. The most notable increment was recorded on the 90th day, with a mean score that was statistically significantly improved from the pre-treatment baseline (p < 0.05). These findings are summarized in Table 3, which delineates the mean STQoL scores at each assessment point alongside the corresponding statistical significance.

**Table 3:** Sore Throat Quality of Life (STQoL) scale scores at baseline and post-treatment intervals *P value was considered significant at < 0.05

STQoL scores	Mean±SD	p-value^*^
Before treatment	22.2 ± 3.2	0.644
After treatment (30th day)	57.9 ± 8.2	0.035
After treatment (60th day)	77.1 ± 13.6	0.019
After treatment (90th day)	93.5 ± 17.3	0.033

## Discussion

The management of CP has historically been impeded by the lack of a universally accepted definition regarding the duration that qualifies a sore throat condition as 'chronic'. In our study, we adopted a pragmatic approach, defining CP as persistent inflammation of the pharyngeal mucosa or a continuous burning or stinging sensation in the throat, which perseveres beyond a three-month period despite pharmacological intervention [12]. This conceptual framework provided a basis for our investigation into the efficacy of various treatment modalities.

Our analysis adhered to a quartet of guiding principles designed to encapsulate the essence of comprehensive patient management. Initially, our primary goal was the reduction of symptom duration and intensity to the point where daily life was no longer adversely impacted. The secondary aim was to achieve enduring symptomatic relief through the application of a multidisciplinary approach that went beyond the symptomatic treatment paradigm typically adopted in general practice [13]. A further aspiration was to preclude both local and systemic sequelae, thereby mitigating the need for extensive pharmacotherapy.

In many clinical scenarios, otolaryngologists are the first point of contact for patients presenting with CP. Standard treatments, if ineffective or if the condition reoccurs, may necessitate a more holistic and innovative treatment strategy. This study proposes such a multidisciplinary approach to CP, suggesting that it may be instrumental in effecting a paradigm shift in the standard of care [13].

Research illuminates the notion that the management of acute pharyngitis, whether of bacterial or viral etiology, is predicated on two critical determinants: the strategic selection of antimicrobial agents and the general health of the immune response [1,14,15]. The enhancement of immune defenses is recognized as a crucial aspect of the therapeutic regime, with an inadequately managed acute episode potentially giving rise to a chronic condition [16]. This highlights the imperative of a detailed patient history and meticulous physical examination. Extensive blood testing, encompassing serum iron, ferritin, vitamin B12, vitamin D, folic acid, HbA1c (for those over 40), and serum IgE levels, offers invaluable insight into the body's systemic health, immune functionality, and its inherent capacity for mucosal repair [17-22].

One must also consider the nasal cavity's condition, as nasal congestion is a significant contributing factor to CP. Structural anomalies, such as nasal septum deviation and turbinate hypertrophy, can be significant etiological factors. These physical barriers impede proper air passage and predispose individuals to mouth breathing, particularly during sleep, leading to a drying out of the pharyngeal mucosa, a state conducive to the development of CP [23,24]. Therapeutic interventions, both surgical and medical, that aim to rectify these anomalies can have profound effects on the pharyngeal environment, potentially reversing the pathogenetic process and restoring the mucosa to its normal state.

Another critical aspect is the role of gastroesophageal reflux in CP. The chronic backflow of stomach acids can cause micro-lacerations in the oropharyngeal mucosa, which not only perpetuates the inflammatory process but is often exacerbated by dietary factors such as the consumption of acidic foods and beverages [25]. Effective management can involve long-term use of oral mouthwashes containing anti-reflux agents, such as sodium alginate and sodium bicarbonate, which can provide symptomatic relief [26].

Allergies, too, play a notable role in the pathophysiology of CP. Mucosal allergic responses, resulting in hypertrophy and irritation of the nasopharyngeal and oropharyngeal tissues, are common culprits. Systemic antihistamine therapy can ameliorate these symptoms, thus constituting an essential component of a comprehensive treatment plan [22].

It is important to underscore that these pathologies do not exist in isolation. Rather, a single patient may present with multiple contributing factors, necessitating a robust, multidisciplinary therapeutic strategy. Such an approach has been validated by the improvements observed in our study, as delineated in Table 2, which offers a clear demonstration of the improvement in STQoL scores following the implementation of our multidisciplinary treatment approach.

In reflecting on standard practices, it is evident that treatments such as mouthwash solutions, which often include benzydamine and chlorhexidine, can be effective. Nevertheless, they are not without drawbacks, including potential taste disorders and dry mouth with long-term use. In contrast, our study's approach sought to circumvent these side effects, offering a comprehensive regimen that extends beyond the mere alleviation of symptoms to target the underlying causative factors of CP [27,28].

In summary, our findings advocate for a nuanced approach to the treatment of CP, one that is predicated on a detailed understanding of the disease's multifactorial etiology and tailored to the individual patient's needs. The significant enhancement in QoL, as evidenced by the STQoL scores, underscores the potential of this method to serve as a new benchmark in CP management. Despite these promising results, the following limitations in this study call for cautious interpretation: first, inherently, causality and generalization to the population at large may not be achievable in a retrospective single-center design; second, incomplete medical records may be a challenge, especially regarding the lifestyle factors of patients or comorbid conditions that could influence disease course and therapy response. Third, even though it is a valuable tool, the STQoL scale supplementation with other objective assessments, such as imaging or standardized physical examination protocols, could further strengthen these conclusions. Lastly, the relatively small sample size underlines the need for larger multi-center prospective studies to confirm findings and refine the proposed multidisciplinary approach.

## Conclusions

Our findings have reiterated a multidisciplinary approach to the treatment of CP, a condition severely affecting the QoL. Our study focused on some critical etiological elements, namely nutrient deficiencies, anatomical nasal obstruction, gastroesophageal reflux, and mucosal allergic response, and thus showed significant improvement in clinical outcomes. These benefits were further supported in the improvements measured using the STQoL scale, with significant changes in the patients' overall well-being post treatment, as compared to conventional symptom-directed therapies. While our findings highlight the potential of a multimodality treatment approach, additional studies are required to further refine and individualize therapy. Future research needs to be directed not only at optimizing long-term patient outcomes but also at offering novel interventions and expanding our knowledge about the pathophysiology of the disease. In this way, health providers are able to continue to ensure efficacy and enhancement of patient satisfaction by further evolving management strategies with a more holistic approach to the management of CP.
